# Occurrence and Prevalence of Insect Pathogens in Populations of the Codling Moth, *Cydia pomonella* L.: A Long-Term Diagnostic Survey

**DOI:** 10.3390/insects4030425

**Published:** 2013-08-02

**Authors:** Gisbert Zimmermann, Alois M. Huger, Regina G. Kleespies

**Affiliations:** Julius Kühn-Institute (JKI), Federal Research Centre for Cultivated Plants, Institute for Biological Control, Heinrichstrasse 243, Darmstadt D-64287, Germany; E-Mails: gisbert.zimmermann@gmx.net (G.Z.); a.huger@web.de (A.M.H.)

**Keywords:** *Cydia pomonella*, diagnosis, insect pathogens, granulovirus, fungi, *Beauveria bassiana*, *Nosema carpocapsae*, host-pathogen-interactions

## Abstract

About 20,550 larvae, pupae and adults of the codling moth, *Cydia pomonella* L., were diagnosed for pathogens during long-term investigations (1955–2012) at the Institute for Biological Control in Darmstadt, Germany. The prevailing entomopathogens diagnosed in these studies were insect pathogenic fungi, especially *Beauveria bassiana* and *Isaria farinosa*, the microsporidium, *Nosema carpocapsae*, the *Cydia pomonella* granulovirus (CpGV), as well as mostly undetermined bacteria. While the CpGV was observed exclusively in larvae and pupae from laboratory colonies or from field experiments with this virus, entomopathogenic fungi were most frequently diagnosed in last instars in autumn and in diapausing larvae and pupae in spring. *B. bassiana* was identified as the major fungal pathogen, causing larval prevalences of 0.9% to 100% (mean, about 32%). During prognostic long-term studies in larvae and adults of *C. pomonella*, *N. carpocapsae* was diagnosed in codling moth populations from various locations in Germany. The mean prevalence generally ranged between 20% and 50%. Experiments revealed that the fecundity and fertility of microsporidia-infected female adults were significantly reduced compared to healthy ones. The results underpin the importance of naturally occurring microbial antagonists and represent a base for further ecological studies on developing new or additional biological and integrated control strategies.

## 1. Introduction

The codling moth, *Cydia pomonella* L. (Lepidoptera, Tortricidae), is a major and nearly worldwide occurring pest insect, mainly of apple trees, necessitating regular control measures. Generally, chemical insecticides are used, but also biological control methods, like pheromone mating disruption, as well as the specific *C. pomonella* granulovirus (CpGV), are applied [1,2,3,4,5,6]. Concerns on safety and the environmental impact of insecticides, as well as the development of resistance have led to an increased use of CpGV. However, recently, some codling moth populations also showed a reduced susceptibility against CpGV products, stimulating the search for further biological control measures [7,8,9,10,11,12,13,14,15,16].

Several naturally occurring microbial antagonists have been reported from *C. pomonella*, including viruses, bacteria, fungi, microsporidia and nematodes [12,17,18,19,20]. So far, CpGV, the bacterium, *Bacillus thuringiensis*, the fungus, *Beauveria bassiana*, the microsporidium, *Nosema carpocapsae*, and, recently, also, nematodes of the genus, *Steinernema*, have been tested in the lab or in the field [7,8,12,15,21,22,23,24,25,26,27,28,29].

Although the codling moth is a key pest in apple orchards, only a few ecological investigations on naturally occurring antagonists, especially insect pathogenic microorganisms, and on their importance as mortality factors in codling moth populations in Germany or Europe were conducted [7,18,25,30,31,32,33,34]. In this context, only a few studies on the causes of mortalities of diapausing larvae during hibernation are available, whereby pathogens could play an important role [34,35]. For example, Zelger *et al*. [36] found a winter mortality of 20–37%, mainly caused by weather conditions and general biotic factors, which are not clearly defined.

Recently, a review and a database on entomopathogens of insects and other arthropods found during long-term diagnostic studies at the Institute for Biological Control, Darmstadt, were published [37,38]. The data reported herein summarizes the type and frequency of pathogens found in 20,550 living, diseased or dead codling moth individuals that were diagnostically examined and the entomopathogens determined (E. Müller-Kögler, fungi: 1955–1974; A. Krieg, bacteria, rickettsiae, viruses: 1955–1989; A.M. Huger, viruses, bacteria, rickettsiae, microsporidia, protists: 1957–1991; G. Zimmermann, fungi: 1974–2005; R.G. Kleespies, viruses, bacteria, rickettsiae, microsporidia, protists: since 1991). As most of the results of these studies are not published and only few of them are briefly outlined in German annual reports of the former Federal Biological Research Centre for Agriculture and Forestry (BBA), a detailed presentation on *C. pomonella* is given here. We believe that these results gained over a long period of time will stimulate further ecological and pathological studies on *C. pomonella*, in order to enrich the understanding of the efficacy of natural microbial control factors within the population development. They also may initiate further approaches to preserve natural ecosystems and to develop new strategies within an integrated control system.

## 2. Material and Methods

### 2.1. Examination and Identification

For diagnostic studies and the identification of entomopathogenic and other microorganisms found in all stages of the codling moth, different microbiological and histopathological methods and techniques were used [22,39,40,41,42,43,44,45,46,47,48,49,50]. First, all specimens were examined microscopically (stereo and light microscope) and, if necessary, also by transmission electron microscope (TEM). In case of an infection by fungi, the larvae and pupae were surface-sterilized with 0.1% hypochlorite and, then, transferred to Petri dishes containing a moist filter paper or a moist cotton plug to stimulate outgrowth of the fungus from the cadaver. For identification, bacteria and fungi were isolated on various media, e.g., Tryptic soy broth (TSB + 1.5% agar; Otto Norwald KG, Hamburg, Germany) or malt peptone agar (3% malt extract, 0.5% peptone, 1.5% agar), respectively, with or without antibiotics (chloramphenicol 50 mg/L). In most cases, a clear identification of bacteria was not conducted. As bacteria are members of the natural gut flora of insects, they can easily be found in dead individuals of *C. pomonella*, even if these had died because of age-related reasons or due to abiotic or other biotic reasons. Fungi were identified according to [51,52,53,54]. Fungal names were adapted, as far as possible, to the actual nomenclature, e.g., the new name of *Paecilomyces farinosus* is *Isaria farinosa* [55,56]. So-called wound or secondary pathogens, including saprophytic fungi, such as *Aspergillus*, *Penicillium* and some Zygomycetes, were only identified to the genus level. As these fungi often are in the gut or on the cuticle of dead insects, their role as insect pathogens for the majority is seldom clear.

To verify CpGV, generally, a fast and simple routine procedure was employed: If wet-mount preparations of fat body tissue strikingly revealed many or even myriads of tiny particles in rapid Brownian movement in phase contrast and dark field, small drops of this material were applied onto Formvar-coated grids, negatively stained by 2.0% phosphotungstic acid and analyzed in TEM-studies. For cyto- and histo-pathological investigations, stained serial sections were employed in light microscope and TEM (see below).

For identifying microsporidia, morphological features of spores and lifecycle stages proved useful when examining squash preparations of host tissues and Giemsa-stained smears. To clarify histo- and cyto-pathological events, stained serial sections of tissues or entire specimens were necessary using the light and TE-microscope. For light microscopy, individuals were fixed with Duboscq-Brazil’s alcoholic Bouin’s and embedded in Histosec (Merck, Darmstadt, Germany). Serial sections at 6–9 µm were stained with Heidenhain’s iron hematoxylin, counterstained with erythrosin and examined in a Leica photomicroscope, model DMRB (Leica, Bensheim, Germany). For electron microscope studies of ultrathin sections, larval tissues were fixed overnight at 4 °C in 3% glutaraldehyde in Veronal buffer (pH 7.2) and postfixed in 1–2% osmium tetroxide in the same buffer for 6–20 hours. After dehydration in ascending ethanol series, tissues were embedded in a 7:3 mixture of butyl and *n*-methymethacrylate [43]. Thin sections, double-stained with uranyl acetate and lead citrate, were examined in a Zeiss EM 9 and a Zeiss EM 902 (since 1991) (Zeiss, Oberkochen, Germany).

The long-term studies on the occurrence and prevalence of *N. carpocapsae* were conducted exclusively by Huger between 1972 and 1978 for larvae and from 1972 until 1990 for the adults [33,57,58]. The specimens were collected in commercial orchards by Mr. Zotzmann from the former Plant Protection Office in Frankfurt/Main using light traps, pheromone traps or from hibernation cages (wire gauze cages fixed on corrugated cardboard bands around apple tree stems to prevent predation e.g. by birds). As genetic investigations have documented that microsporidia belong to fungi (e.g., [59,60]), they are mentioned here as Fungus/Microsporidium.

### 2.2. Experiments with Nosema carpocapsae

The effect of the microsporidium *N. carpocapsae* on the fecundity and fertility of *C. pomonella* was examined in six laboratory experiments and, so far, only briefly published [25]. Ten female and 20 male adults, deriving from a healthy and a microsporidia-infected laboratory-reared colony were used in each bioassay. Ten groups of one female and 2 males were kept in a plastic box (ø, 6 cm, height, 5 cm) that was lined inside with a thin layer of foamy material in order to concentrate egg-laying onto the clean, smooth and transparent lid. The boxes were stored in an incubator at 23 °C and permanent light sources on each side. The number of eggs laid per female and the number of hatched larvae were determined. For statistical analysis of the data on fecundity and fertility, the *SAS System for Windows, Version 9.1* was used. Significant differences between the means were calculated using the glm-procedure (general linear models) and the Student-Newman-Keuls-test (*p* ≤ 0.05) and angular transformed data (fertility).

## 3. Results

### 3.1. General Information on Accessions

Altogether, 89 accessions of living and/or dead larvae, pupae and adults of *C. pomonella* mostly from field populations in commercial orchards were examined. They included about 20,550 individuals, *i.e.*, 8,600 larvae, 800 pupae, 10,200 adults, as well as a mixture of larvae and pupae (n = 900) and of all life stages (n = 50) that could not be separated clearly. About 90% (n = 79) of all accessions were from Germany, one each from Italy and Israel, two from Austria and six from Switzerland. Accessions were mainly sent by plant protection services, some of them also by companies maintaining a codling moth colony. Most larvae were last instars or diapausing larvae collected in autumn or spring, respectively, from corrugated cardboard bands. Adults were caught in light traps or in pheromone traps or were collected from hibernation cages and examined for microsporidia and other pathogens during prognostic studies (51 accessions, 57.3%). Furthermore, 25 accessions (28.1%) contained larvae, pupae and adults of codling moth laboratory colonies.

### 3.2. Pathogens, Other Microorganisms and Nematodes of C. pomonella (Larvae, Pupae, Adults) from Field Populations

**Figure 1:**
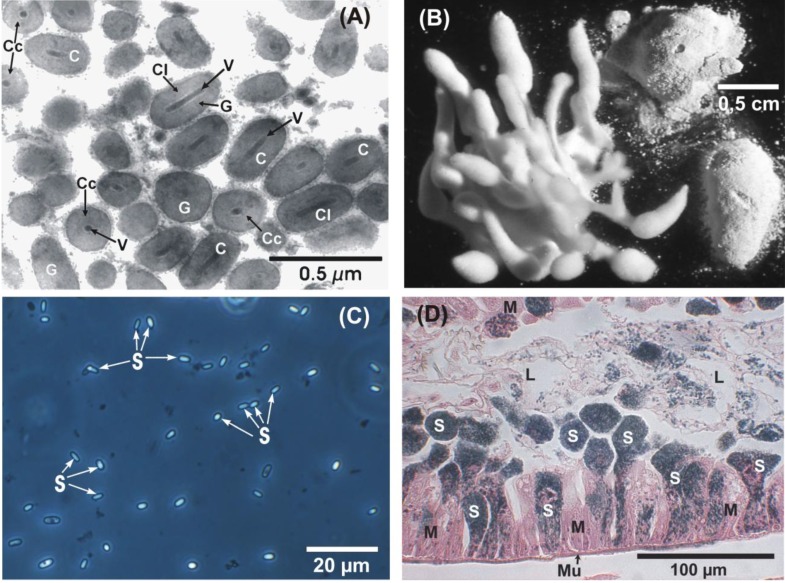
Important entomopathogens of the codling moth, *Cydia pomonella.* (**A**) Thin section of granulovirus (CpGV) with capsule-shaped virus occlusion bodies (C), formed by proteinaceous granulin (G). Each virus capsule (C) harbors one rod-shaped virion (V). Longitudinal sections (Cl) and cross sections (Cc) of virus capsules (transmission electron microscope); (**B**) Different morphologies of the entomopathogenic fungus, *Beauveria bassiana*, on three codling moth larvae (stereomicroscope); (**C**) Spores (S) of the microsporidium, *Nosema carpocapsae* (phase contrast microscope); (**D**) Longitudinal section of larval midgut epithelium (M) with heavy infection by *N. carpocapsae*. Rounded batches of the dark colored spores (S) are expelled from the cylindrical epithelium cells into the midgut lumen (L). Mu, muscularis of the midgut. (Bright field microscope, staining: Hematoxylin Heidenhain—Erythrosin).

In specimens received from field populations, insect pathogenic and other fungi represented the major group of diagnosed organisms, followed by bacteria, microsporidia (mostly *N. carpocapsae*), the granulovirus, CpGV, and nematodes (Table 1 and Figure 1). Fungi were mostly diagnosed in last instars and in diapausing larvae in autumn and spring, respectively, and often also mixed infections of two or three species were observed. The predominant entomopathogenic species was *B. bassiana*, followed by *I. farinosa* (syn. *P. farinosus*), *Hirsutella* spp., *Lecanicillium* spp. (syn. *Verticillium lecanii*), *Isaria fumosorosea* (syn. *Paecilomyces fumosoroseus*) and *Metarhizium anisopliae*. *B. bassiana* and *I. farinosa* were also detected frequently in mixed infections, and in some cases, they were hyperparasitized by the ascomycete, *Syspastospora parasitica* (syn. *Melanospora parasitica*) [21,61]. Other genera, such as *Alternaria*, *Aspergillus*, *Cephalosporium*, *Cladosporium*, *Fusarium*, *Mucor* or *Penicillium*, also occurred relatively often. Whether these fungi are true entomopathogens is not yet clear. Bacteria were mostly diagnosed in dead specimens of all developmental stages of *C. pomonella*. Unidentified spore-formers and *Serratia* spp. were predominant. The microsporidium, *N. carpocapsae*, was found in larvae and pupae, as well as in living and dead adults, where it was diagnosed as the only pathogen. Occasionally, a double infection with the CpGV, some fungi, bacteria and unidentified nematodes was observed. CpGV was detected exclusively in dead larvae and pupae.

**Table 1:** Overview on diagnosed pathogens, other microorganisms and nematodes in *C. pomonella* (larvae, pupae, adults) detected in the Institute for Biological Control (1955–2012); (modified from [37]; AT, Austria; CH, Switzerland; DE, Germany).

Pathogen-Group	Pathogens/Microorganisms/Nematodes	Origin (country)
Viruses	Granulovirus	CH, DE
	Granulovirus + *Nosema carpocapsae*	CH, DE
Bacteria	Bacteria, unidentified	DE
	Bacteria (spore-formers), unidentified	DE
	*Serratia liquefaciens*	DE
	*Serratia* sp.	DE
	*Hafnia alvei* + *Serratia* sp. + *Pseudomonas* sp.	DE
	*Serratia* sp. + *Nosema carpocapsae*	DE
	*Bacillus cereus* + nematodes, unidentified	DE
Fungi	*Alternaria* sp.	DE
	*Aspergillus flavus*	DE
	*Aspergillus* sp.	AT, DE
	*Beauveria bassiana*	AT, DE
	*Beauveria bassiana*, hyperparasitized by *Syspastospora parasitica* (*Melanospora parasitica*)	DE
	*Beauveria* sp.	AT
	*Cephalosporium* sp.	AT, DE
	*Cladosporium* sp.	AT
	*Fusarium avenaceum*	AT
	*Fusarium* sp.	AT, DE
	*Hirsutella gigantea*	AT
	*Hirsutella* sp.	AT, CH, DE
	*Hirsutella subulata*	AT
	*Isaria farinosa*	AT, DE
	*Isaria farinosa* hyperparasitized by *Syspastospora parasitica* (*Melanospora parasitica*)	AT, DE
	*Isaria fumosorosea*	DE
	*Lecanicillium* sp. (*Verticillium lecanii*)	DE
	*Metarhizium anisopliae*	AT
	*Mucor* sp.	AT, DE
	*Penicillium* sp.	AT, DE
	*Verticillium* sp.	DE
	**Mixed infections**	
	*Alternaria* sp. + *Cephalosporium* sp.	DE
	*Alternaria* sp. + *Fusarium* sp.	DE
	*Aspergillus* sp. + *Fusarium* sp.	AT, DE
	*Aspergillus* sp. + *Mucor* sp.	AT
	*Aspergillus* sp. + *Fusarium* sp. + *Penicillium* sp.	AT
	*Beauveria bassiana* + bacteria, unidentified	AT
	*Beauveria bassiana* + *Aspergillus* sp.	AT
	*Beauveria bassiana* + *Cephalosporium* sp.	DE
	*Beauveria bassiana* + *Cladosporium* sp.	AT
	*Beauveria bassiana + Hirsutella* sp.	DE
	*Beauveria bassiana* + *Mucor* sp.	AT
	*Beauveria bassiana + Isaria farinosa*	DE
	*Beauveria bassiana* + *Penicillium* sp.	DE
	*Beauveria bassiana* + *Aspergillus* sp. + *Hirsutella* sp.	AT
	*Beauveria bassiana* + *Mucor* sp. + *Penicillium* sp.	AT
	*Beauveria bassiana* + *Penicillium* sp. + *Aspergillus* sp.	AT
	*Hirsutella* sp. + *Aspergillus* sp.	AT
	*Hirsutella* sp. + *Mucor* sp.	AT
	*Isaria farinosa* + *Alternaria* sp.	DE
	*Isaria farinosa + Fusarium* sp.	DE
	*Isaria farinosa* + *Penicillium* sp.	AT
	*Isaria farinosa* + *Beauveria bassiana* + *Penicillium* sp.	AT
	*Isaria farinosa* + nematodes, unidentified	DE
	*Paecilomyces* sp. + *Alternaria* sp.	DE
	*Paecilomyces* sp. + *Beauveria* sp.	DE
	*Paecilomyces* sp. + *Mucor* sp.	DE
	*Paecilomyces* sp. + *Verticillium* sp.	DE
	*Penicillium* sp. + *Alternaria* sp.	DE
	*Penicillium* sp. +*Aspergillus* sp.	DE
	*Penicillium* sp. +*Cephalosporium* sp.	DE
	*Penicillium* sp. + *Mucor* sp.	AT, DE
	*Verticillium* sp. + *Alternaria* sp.	DE
	*Verticillium* sp. + *Penicillium* sp.	DE
Fungi/Microsporidia	*Nosema carpocapsae*	CH, DE
	*Nosema carpocapsae* + nematodes, unidentified	DE
	*Nosema carpocapsae +* fungi, unidentified	DE
	Microsporidia, unidentified + bacteria, unidentified	CH
Nematodes	Nematodes, unidentified	DE

### 3.3. Pathogens and Other Microorganisms of C. pomonella (Larvae, Pupae, Adults) from Laboratory Colonies

In 28% of all accessions containing larvae, pupae and adults from laboratory colonies of *C. pomonella*, mostly infections by CpGV and *N. carpocapsae*, as well as by bacteria of the genera, *Serratia* and *Pseudomonas*, were identified, besides undetermined bacteria (Table 2). In one accession, *B. bassiana* and *I. farinosa* were diagnosed in larvae. It is assumed that these larvae already were infected when brought to the laboratory.

**Table 2:** Diagnosed pathogens and other microorganisms in *C. pomonella* (larvae, pupae, adults), from laboratory colonies.

Pathogen-Group	Pathogens/Microorganisms	Accessions (n)
Viruses	Granulovirus	4
	Granulovirus + *Nosema carpocapsae*	1
	Granulovirus + microsporidia, unidentified	3
Bacteria	Bacteria, unidentified	4
	*Serratia liquefaciens*	1
	Bacteria, unidentified+ *Serratia* sp.	1
	Bacteria, unidentified + microsporidia, unidentified	1
	*Serratia* sp. + *Pseudomonas* sp.	1
Fungi	*Beauveria bassiana*	1
	*Isaria farinosa*	1
Fungi/Microsporidia	*Nosema carpocapsae*	6
	*Nosema carpocapsae* + *Serratia* sp.	1

### 3.4. Occurrence and Prevalence of the Most Frequent Pathogens

The most frequent entomopathogens found in larvae, pupae or adults of all examined *C. pomonella* were the CpGV (10.1%), *B. bassiana* (23.6%) and *N. carpocapsae* (57.3%). Therefore, some special aspects on their occurrence and prevalence in *C. pomonella* are presented here.

#### 3.4.1. *Cydia pomonella* Granulovirus (CpGV)

CpGV was exclusively found in larvae and pupae from apparently inadvertently contaminated laboratory colonies or from field experiments, where the CpGV was applied before as plant protection measure (infestation rates 1.0–100%). Thus, no evidence for a natural occurrence of CpGV was obtained during our studies.

#### 3.4.2. *Beauveria bassiana*

In nearly all specimens of diseased or dead last instars in autumn or in diapausing larvae, as well as in pupae in spring, the fungus, *B. bassiana*, was detected during the whole period of diagnostic survey in various populations and different areas in Germany and one in Austria (Table 3). The number of individuals per accession was between three and 2,355. This documents that *B. bassiana* is the most important entomopathogenic fungus of *C. pomonella* under natural conditions irrespective of the year or origin. As mentioned, mixed infections with other entomopathogenic fungi, mainly *I. farinosa* and *Hirsutella* sp., as well as with other species were observed. In all accessions with specimens being only infected by *B. bassiana*, the prevalence of examined larvae and pupae ranged between 0.9% and 100%; on average, about 32%.The rate of mixed infections with other fungi was 0.3% to 5.4%; in single cases, up to 25%.

**Table 3:** Occurrence and prevalence of the entomopathogenic fungus, *Beauveria bassiana* (*B.ba.*), in *C. pomonella* (larvae and pupae; the origin mostly refers to the address of the sender and not always to the location of the codling moth).

Origin	Year	Results	Infestation in % and total number of individuals examined (n)
**Austria**			
Kronberg	1971	*B.ba.*	**36.9** (111)
		*B.ba.* + *Aspergillus* sp.	**1.8**
		*B.ba.* + *Aspergillus* sp. + *Hirsutella* sp.	**0.9**
		*B.ba.* + bacteria, unidentified	**0.9**
		*B.ba.* + *Cladosporium* sp.	**1.8**
		*B.ba.* + *I. farinosa* + *Penicillium* sp.	**1.8**
		*B.ba.* + *Mucor* sp.	**1.8**
		*B.ba.* + *Mucor* sp. + *Penicillium* sp.	**0.9**
		*B.ba.* + *Penicillium* sp. + *Aspergillus* sp.	**1.8**
	1972	*B.ba.*	**59.9** (2,355)
**Germany**			
Dossenheim	1983	*B.ba.*	**0.9** (1,954)
		*B.ba.* + *Paecilomyces* sp.	**0.3**
Frankfurt	1955	*B.ba*.	**88.2** (17)
	1973	*B.ba.*	**87.5** (24)
		*B.ba.* *+ I. farinosa*	**4.2**
		*B.ba.* + *Penicillium* sp.	**4.2**
	1974	*B.ba.*	**87.8** (74)
		*B.ba.* + *Syspastospora parasitica*	**5.4**
	1975	*B.ba.*	-
		*B.ba.* + *Cephalosporium* sp.	
		*B.ba.* + *I*. *farinosa*	
Heidelberg	1957	*B.ba.*	**42.8** (14)
Mainz	1967	*B.ba.*	-
Neustadt/Meckenheim	1955/1	*B.ba.*	*ca* **. 20** (*ca*. 25)
	1955/2	*B.ba.*	*ca* ** . 75** (*ca*. 52)
		*Mucor* sp. on *B.ba*.	*ca* **. 25**
Neustadt/Weisenheim	1955	*B.ba.*	**53.3** (75)
Offenbach	1973	*B.ba.*	**100** (23)
Stuttgart	1957	*B.ba.*	**29.4** (17)
	1973	*B.ba.*	**8.2** (49)
	1975	*B.ba.*	**30.0** (40)
		*B.ba.* + *I*. *farinosa*	**2.5**
	2002	*B.ba.*	**19.2** (52)
		*B.ba.* + *Hirsutella* sp.	**1.9**
	2004	*B.ba.*	**9.2** (488)
	2005	*B.ba.*	**2.4** (454)
Wiesbaden	1958	*B.ba.*	**100** (5)

#### 3.4.3. *Nosema carpocapsae* and Its Effects on Fecundity and Fertility of *C. pomonella*

In more than 50% of all examined accessions, the microsporidium, *N. carpocapsae*, was identified. Most of these accessions (39.1%) were received within a prognostic survey on the prevalence of *N. carpocapsae* in common field populations of the codling moth. This pathogen was mainly found in last instars in autumn, as well as in adults caught in the field by light traps pheromone traps or collected from hibernation cages. *N. carpocapsae* may also occur in mixed infections with CpGV, unidentified bacteria or nematodes in laboratory colonies of the codling moth (see Table 1, Table 2). Larvae and adults from field populations originated from Hessen (Hassia) (Darmstadt, Frankfurt, Kriftel, Niederhofheim and Geisenheim), from Bayern (Bavaria) (Deutenkofen, Erlabrunn, Neuhaus, Igensdorf, Eichelsdorf, Pittersberg, Mallersdorf, Uffenheim and Weihenstephan) and from Baden-Württemberg (Baden-Wurttemberg) (Dossenheim, Heidelberg, Ludwigsburg and Karlsruhe Augustenberg). The diagnosed infestation rates of larvae (seven years, 1972–1978) and adults (19 years, 1972–1990) from various locations and regions are presented in Table 4, Table 5, respectively. The data show that *N. carpocapsae* was widely distributed at that time mainly in lower areas of the Rhein-Main-District. Last instars (L_5_) examined between 1972 and 1978 achieved high prevalences by *N. carpocapsae* in the first years in Frankfurt-Liederbach (62.0%), Kriftel (57.5%), Niederhofheim (up to 52.8%) and Heidelberg-Kirchheim (74.0%), with a certain decrease in subsequent years (Table 4). In diagnosed adults (Table 5), the infestation rates during 16 years in Frankfurt, for example, ranged between 48.2% in 1975 and 33.6% in 1990 (minimum: 33.0%/1983; maximum: 59.7%/1978), during five years in Geisenheim, between 49.8% in 1973 and 51.7% in 1977 (minimum: 45.5%/1976; maximum: 59.9%/1974) and during 15 years in Kriftel between 30.4% in 1973 and 30.8% in 1987 (minimum: 16.8%/1982; maximum: 57.3%/1985). Thus, generally, rather constant prevalences were noticed over the years. In Bayern, the number of infested adults caught in light traps was between 0% and 33.3%, but altogether lower, as compared to Hessen. In the adults received from Baden-Württemberg, only one accession from Heidelberg showed a high prevalence of 50.6% (1975).

**Table 4:** Occurrence and prevalence of the microsporidium, *Nosema carpocapsae*, in larvaeof *C. pomonella*, from various regions in Germany (Hessen, Bayern and Baden-Württemberg; 1972–1978).

Origin	Year—Infestation in % and number of individuals (n)						
Hessen (Rhein-Main area)	1972	1973	1974	1975	1976	1977	1978
Billings			0 (20)				
Darmstadt	57.1 (7)						
Frankfurt-Liederbach		62.0 (50)			41.5 (94)		
Kriftel		57.5 (80)	37.0 * (309)	30.8 * (100)	17.9 * (140)	36.8 * (36)	
Niederhofheim		35.3 (122)	43.4 * (237)	40.0 (30)	22.9 (70)		
**Bayern**							
Pittersberg					29.8 (94)		
**Baden-Württemberg**							
Dossenheim			26.3 * (103)	18.1 * (791)	17.3 (110)		
Heidelberg-Kirchheim		74.0 (49)	50.3 * (79)	33.3 (30)			
Karlsruhe-Augustenberg						3.0 (30)	20.0 (35)
Weinheim			23.3 (30)				

* Means from several trappings.

**Table 5:** Occurrence and prevalence of the microsporidium, *Nosema carpocapsae*, in adults of *C. pomonella*, from various regions in Germany (Hessen, Bayern and Baden-Württemberg) captured by light traps and pheromone traps, as well as from hibernation cages (1972–1990).

Origin	Year–Infestation in % and number of individuals (n)																	
Hessen (Rhein-Main area)	1972	1973	1974	1975	1976	1977	1978	1979	1980	1981	1982	1983	1984	1985	1986	1987	1989	1990
Darmstadt (light trap)	23.9 (117)						27.9 (147)	26.0 (100)										
Frankfurt (light trap)				48.2 (170)	55.5 (321)	44.0 (275)	59.7 (139)	34.4 (206)	44.4 (187)	38.5 (377)	46.6 (253)	33.0 (264)	42.4 (184)	46.8 (171)	37.2 (204)	34.4 (90)	43.5 * (361)	33.6 (235)
Geisenheim (light trap)		49.8 * (253)	59.9 * (372)	55.4 (186)	45.5 (110)	51.7 (29)												
Kriftel (hibernation cages)		50.0 (12)	50.0 (28)	45.0 (20)	51.5 (33)		0 (1)											
Kriftel (light trap)		30.4 * (303)	31.7 (202)	45.4 (77)	38.1 (189)	22.4 (112)	29.5 (61)	31.7 (63)	29.0 (48)	22.3 (94)	16.8 (125)	18.7 (112)	22.9 (135)	57.3 (75)	25.8 (124)	30.8 (39)		
Kriftel (pheromone trap)				30.7 (319)	35.5 (284)	18.1 (343)	25.3 (162)	25.5 (153)	23.7 (156)									
Langensel-bold (pheromone trap)				38.0 (50)														
Nordenstadt (light trap)								0 (13)										
**Bayern**																		
Deutenhofen (light trap)		20.5 * (259)	24.0 (50)	23.5 (51)														
Erlabrunn (light trap)		6.3 (16)	20.0 (15)	11.9 (76)	11.7 (170)													
Neuhaus (light trap)		0 (18)	0 (11)	0 (15)	33.0 (3)													
Igensdorf (light trap)		0 (3)																
Eichelsdorf (light trap)		0 (35)	10.0 (10)															
Mallersdorf (light trap)			27.3 (11)	33.3 (3)														
Uffenheim (light trap)			33.3 (3)	14.3 (21)	19.0 (21)													
Weihen-stephan (light trap)					100 (5)													
**Baden-Württemberg**																		
Dossenheim (light trap)			28.4 * (123)	24.1 * (112)														
Dossenheim (hibernation cages)												20.8 * (120)						
Heidelberg (pheromone trap)				50.6 (16)														
Ludwigsburg (pheromone trap)				0 (27)														

* Means from several trappings.

**Figure 2:**
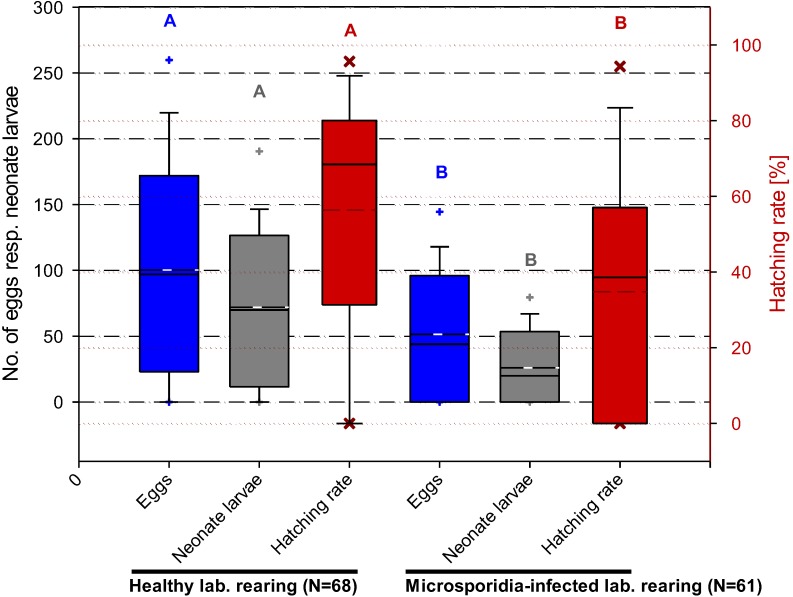
Comparison of fecundity and fertility data from healthy and microsporidia-infected codling moth adults. Box plot with median (solid line) and arithmetic mean (- - -) and the 5 and 95% percentile (+), respectively. Means of the eggs and neonate larvae and the angular transformed values of the hatching rate with diverse letters are significantly different (Student-Newman-Keuls test, *p* ≤ 0.05).

Experiments on the effect of a microsporidian infection on the fecundity and fertility of *C. pomonella* revealed significant differences between healthy and infested female adults (Figure 2). The mean egg number of healthy females was 105 per individual, compared to only 64 of females infected by *N. carpocapsae*. Similarly, the hatching rate of egg samples of healthy females was 64% compared to only 46% of those laid by infected females (see, also, [25]).

## 4. Discussion

The diagnostic studies represent an overview on entomopathogenic and other microorganisms, including nematodes, found in larvae, pupae and adults of the codling moth over a longer period of time. Except for *N. carpocapsae*, the investigations were not conducted systematically by routine examination of field populations, but irregularly whenever codling moth specimens were sent to the Institute for Biological Control for diagnosis of their diseases.

The most frequently diagnosed entomopathogens were the codling moth granulovirus (CpGV), various fungi, especially *B. bassiana* and *I. farinosa*, and *N. carpocapsae*. Long-term prognostic studies on the prevalence of *N. carpocapsae* in larvae and adults in various areas of Germany document the state and development of infestation in different field populations.

CpGV was only found in larvae and pupae from laboratory colonies and in field populations where the virus was applied for plant protection. The reason for the occurrence of CpGV in laboratory reared larvae is not always clear. Probably, they are contaminated accidentally by handling. Thus, a natural occurrence of CpGV, so far, is not proven in Germany, Austria or Switzerland. Certainly, it is difficult to find infected larvae in the field, as they rapidly decay. Whether a propagation of CpGV from virus-treated orchards to other untreated populations occurs, e.g., by contaminated flying insects, by birds or by atmospheric conditions, is scarcely investigated [62,63,64]. The first detection of CpGV was in 1963 in an orchard in Mexico [65].

The role of the observed unidentified bacteria, spore-formers or *Serratia* spp. is unclear. They may be part of the normal gut flora, but some of them also may be pathogens. During epizootics in different laboratory colonies of *C. pomonella* in Poland, several strains of the well-known pathogen, *B. thuringiensis*, were isolated [20]. In the present study, however, *B. thuringiensis* was not found. In any case, further investigations on the effect of these microorganisms on individuals and on field populations of *C. pomonella* are necessary.

Entomopathogenic fungi and, especially, *B. bassiana* and *I. farinosa*, were the major and most frequent group of pathogens diagnosed in last instars and diapaused larvae and pupae. The frequent occurrence and the high prevalence of *B. bassiana* show that this species is an important naturally occurring mortality factor, mainly in hibernating populations, which obviously is permanently present in orchard ecosystems. This fungus has often been found in *C. pomonella* and other tortricids [7,18,30,31,32,34,66]. In an orchard in Austria (Steiermark), Russ [31] observed an outbreak of *B. bassiana* in diapausing larvae with prevalences of 77%, while in other more dry locations, only 1.3% and 2.5% of the larvae were infected. During investigations on diapausing larvae and in pupae of the codling moth in the south of Sweden, besides different parasitoids, mainly *B. bassiana* (34.4%) and *I. farinosa* (29.5%) were detected [34]. In Nova Scotia, Canada, however, Jacques and MacLellan [66] found that fungi only killed 1.7%, with a maximum of 10% of diapausing larvae of *C. pomonella* populations. According to our knowledge, no detailed investigations on the role of entomopathogenic fungi as natural mortality factors and their impact on field populations in Germany and other European countries are available. Furthermore, it is unknown where and when larvae of the codling moth become infected by *B. bassiana* or *I. farinosa* in apple trees, *i.e.*, also studies on the ecology of these two fungi in the field are necessary to clarify their role as natural mortality factors. In addition, investigations on these fungi as pathogens of codling moth larvae from conventionally and ecologically treated apple orchards of different regions would be important to determine possible impacts of other plant protection treatments (e.g., fungicides or herbicides) on the prevalence of these antagonists.

The most frequently identified pathogen of codling moth larvae and adults was *N. carpocapsae*, which causes a chronic disease. For the first time, data are presented on the occurrence and prevalence of *N. carpocapsae* in *C. pomonella* populations from various locations of Germany over a long period of time. It was demonstrated that this microsporidium causes prevalences of living specimens up to *ca*. 70%. The average larval infestation levels in various codling moth populations from different areas in Germany (Hessen, Bayern, Baden-Württemberg) ranged from about 20% to 50% (see, also, [25,33]), which documents a relatively prevalent occurrence at that time. Similar infection levels were ascertained in adults caught in light traps in hessen over 18 years. This microsporidium was already observed in Germany in 1955 and, then, as documented here, in 1972 and in the following years in larvae, pupae and, particularly, also, in adults of *C. pomonella* (see, also, [33,57]). This is remarkable, as *N. carpocapsae* was reported for the first time in North America only in 2001 [8]. Huger [25,33] demonstrated that the chronic infection is transmitted transovarially from infected females to the progeny. There was no significant difference in the infestation rate of female and male adults by *N. carpocapsae* [67].

Significant differences in the fecundity and fertility of healthy and infected adults of *C. pomonella* were observed (Figure 1; [25]). Thus, in the infected batch, an average decrease of the number of hatched egg larvae by 56% was determined. Therefore, it can be concluded that, depending on the natural prevalence, *N. carpocapsae* also may have an appreciable impact in reducing field populations. However, data on the actual infestation rates of codling moth populations by this microsporidium are not available. Similar observations have been published by Andreadis [68] and Lewis *et al*. [69] for populations of the European corn borer, *Ostrinia nubilalis*, infected by *Nosema pyrausta*, resulting in a reduced fertility between 30% and 50%.

Reduced fertility of adults caused by *N. carpocapsae* may also lead to some problems in laboratory reared colonies [25,70]. In contrast, Siegel *et al*. [8] did not find any effect of a North American isolate of *N. carpocapsae* on the fecundity of females; however, a higher mortality and longer developmental period compared to healthy larvae and pupae were noticed. Furthermore, they showed that the first hatched larvae from one egg mass nearly were uninfected. Negative effects of microsporidia on the rate of progeny were also documented for the European corn borer, *O. nubilalis* infected by *N. pyrausta* [71] and the gypsy moth, *Lymantria dispar*, infected by *Vairimorpha disparis* and *Nosema lymantriae* [72,73,74,75,76], where an increase in the mortality of larvae was noted. No effect of *N. carpocapsae* on the mortality of diapausing larvae of *C. pomonella* was observed [33], while there was a clear correlation between the mortality of diapausing larvae of *O. nubilalis* and their infestation by *N. pyrausta* [77].

In addition to these direct effects of *N. carpocapsae* on its host, *C. pomonella*, various interactions between this microsporidium and other antagonists are known. Experiments on the co-occurrence of *N. carpocapsae* and CpGV in laboratory colonies revealed that microsporidia-infected individuals are six-times less susceptible to the CpGV compared to healthy ones [78]. The effects of such double infections to codling moth populations under field conditions are unknown. Generally, microsporidia are rather selective entomopathogens with a small host range. However, in a laboratory colony of the codling moth parasitoid, *Ascogaster quadridentata* (Hymenoptera, Braconidae), it was observed that *N. carpocapsae* not only infects *C. pomonella*, but also the egg-parasitoid itself, thus resulting in a collapse of the rearing [79,80]. Furthermore, an infection of the egg-parasitoid, *Trichogramma evanescens*, by *N. carpocapsae* was proven, causing a reduction of the parasitic capacity of the microsporidia-infected *Trichogramma* females [81]. Possible interactions of *N. carpocapsae* with these parasitoids and their effect on codling moth populations in the field and their ecological relevance are not yet investigated.

Diagnostic studies on the occurrence and prevalence of naturally occurring insect pathogens of *C. pomonella* are important, also with regard to their possible use as biocontrol agents. For example, a strain of *M. anisopliae* (M.a. 43, JKI fungus collection) isolated from a codling moth larva in 1971 originating from Austria was successfully used under the strain names, F52 or BIPESCO 5, against other pest insects, e.g., the black vine weevil, *Otiorhynchus sulcatus* (Coleoptera: Curculionidae), and was the active ingredient of the former mycoinsecticide, “BIO 1020”, of the company, BAYER [82] or against white grubs of the garden chafer [83].

So far, only the CpGV is commercialized for codling moth control (e.g., [3]), but recently, also, entomoparasitic nematodes (*Steinernema carpocapsae* and *S. feltiae*) were tested successfully [7,15,27,29,84,85]. Formerly, also, the fungus, *B. bassiana*, was tested in laboratory and field experiments [7,21,23,24,26]. As already suggested [21] and according to our findings of the fungus in the last larval or diapausing instars, an application on branches and stems in late summer or autumn seems to be most promising, as well as a soil application under trees to infect overwintering larvae and to increase the antagonistic potential. In contrast to these insect pathogens, *N. carpocapsae* was never used as a biocontrol agent against *C. pomonella*. However, other microsporidia, such as *Nosema pyrausta*, were successfully introduced in field populations of the European corn borer, *Ostrinia nubilalis* [68,86,87,88]. Furthermore, the microsporidia, Nosema sp. and Vavraia sp., could be established in populations of the gypsy moth, *Lymantria dispar* [89].

## 5. Conclusions

The results of these long-term diagnostic and other investigations show that *C. pomonella* is attacked by several entomopathogens that are important mortality factors. However, the knowledge on the impact of these microbial antagonists and on possible interactions with other organisms in field populations is scarce. Therefore, the data presented here may serve as a useful base for further systematic studies on the ecology, natural occurrence and prevalence of these entomopathogens in codling moth populations. With regard to increasing resistance problems and the aimed reduction of chemical pesticides, an assessment of their potential and of the possibilities for their conservation and augmentation in the field is important with respect to the development of new strategies for biological or integrated control of *C. pomonella*.
